# En Face Optical Coherence Tomography Imaging Ellipsoid Zone Regeneration in Laser-Induced and Solar Maculopathies

**DOI:** 10.1155/2019/3849871

**Published:** 2019-11-21

**Authors:** Jeanne M. Gunzinger, Katrin Fasler, Daniel Barthelmes, Peter Maloca, Pascal W. Hasler, Christian Böni, Sandrine A. Zweifel

**Affiliations:** ^1^Department of Ophthalmology, University Hospital Zurich, Zurich 8091, Switzerland; ^2^Institute of Molecular and Clinical Ophthalmology Basel (IOB), Basel, Switzerland; ^3^Department of Ophthalmology, University Hospital Basel, Basel 4056, Switzerland; ^4^OCTlab, Department of Ophthalmology, University Hospital Basel, Basel, Switzerland; ^5^Moorfields Eye Hospital, London, UK

## Abstract

The purpose of the study was to analyze imaging findings in spectral domain en face optical coherence tomography (SD OCT) in patients with laser‐induced and solar maculopathies focusing on the possible regeneration of the ellipsoid zone. In a retrospective case series of 3 patients (4 eyes) with solar maculopathy and 2 patients (3 eyes) with laser‐induced maculopathy who underwent a comprehensive ocular examination, ellipsoid zone (EZ) was segmented from SD OCT data. Evaluation of EZ in en face OCT revealed a hyporeflective lesion surrounded by a hyperreflective border. The area of EZ alteration was measured manually in en face OCT. All patients showed partial EZ regeneration. Mean EZ alteration decreased from 0.12 mm^2^ (range: 0.05–0.32) at baseline to 0.07 mm^2^ (range: 0.01–0.22) at last follow‐up (*p* = 0.018, mean follow‐up: 372 days; range: 115–592). Mean best visual acuity (BVA) improved from 20/36 at baseline to 20/30 (*p* = 0.018). In conclusion, en face OCT imaging clearly delineated the area of EZ alteration in patients with laser‐induced and solar maculopathies. Follow-up showed significant reformation of the EZ as well as improvement of BVA.

## 1. Introduction

Laser‐ induced maculopathies as well as solar maculopathy are two rather rare photo‐induced occurrences. Ex vivo histological examinations of solar retinopathy mainly show changes in the outer retina, more precisely in foveal and parafoveal photoreceptor cells (containing photo pigments) and retinal pigment epithelium (RPE, containing melanin, lipofuscin and retinoids) [1]. The parafoveal rod cells have pyknotic nuclei as a sign of inevitable death of these cells. Retinal pigment epithelium cells also show slight structural anomalies, suggesting possible cell death and detachment from Bruch's membrane. Furthermore, there are degenerative changes and loss of melanin granules of the RPE [2]. Minor damages to the RPE can be repaired by local division and/or cell sliding [1].The rather transparent cells of the inner retina are not directly involved in laser‐induced or solar maculopathy [3].

The rapid development of optical coherence tomography (OCT) technology allows in vivo examinations of the different retinal and choroidal structures. Typical findings in patients with solar maculopathy include focal defects in the hyperreflective layers corresponding to the ellipsoid zone (EZ), the interdigitation zone, and the RPE [4, 5, 6]. These defects are mostly surrounded by a distinctive hyperreflective ring in en face OCT, presumably consisting of cellular debris [7]. The defect in the hyperreflective bands of the outer retina often disappears within weeks, which underlines the possibility of RPE recovery or regeneration mentioned above. Laser‐induced maculopathies show similar patterns, but can present more extensive alterations including full thickness macular holes and/or retinal haemorrhage [8, 9]. Regeneration of the EZ after laser‐induced or solar maculopathy has also been documented, although several authors described persistent defects and concluded that this is due to an irreparable degeneration (as photoreceptors are post mitotic) [5, 6].

Laser‐induced and solar maculopathies are both photic retinopathies which, although involving different pathogenetic types, often present similar clinical features and regeneration patterns. To further improve knowledge about the changes and regeneration of the EZ in both of these entities, we conducted a quantitative analysis using en face spectral domain (SD) OCT. We analysed short‐term and long‐term findings of two patients with laser‐induced and three patients with solar maculopathy, who presented themselves within one week after the incident. Additionally, we looked for similarities and differences between laser‐induced and solar maculopathies.

## 2. Materials and Methods

This retrospective chart review was approved by the local ethics committee. Five patients (seven involved eyes) who presented themselves at the emergency clinic of the Department of Ophthalmology of the University Hospital of Zurich with visual complaints due to sun or laser exposure within the prior week were identified. Each patient underwent history taking and extensive ocular examination including best visual acuity (BVA) using glasses and/or pinhole, anterior segment examination, dilated ophthalmoscopic examination of the macula and peripheral retina, OCT imaging, and fundus photography. The SD OCT images were obtained with the Heidelberg Spectralis (version 1.9.13.0) and analyzed with the Heidelberg software (Spectralis Viewing Module 6.5.2.0; Heidelberg Engineering, Heidelberg, Germany). In addition to the standard imaging protocol (Scan area was at least a 15 × 25° rectangle centered on the macula covered by 49 B‐scans) a dense raster scanning protocol (with a pattern size of at least 15 × 5 and a distance between B‐Scans of 11 *μ*m or 33 *μ*m) or so‐called transverse imaging was acquired in every patient. Automated OCT layer segmentation was checked and adjusted manually where necessary. Area, largest diameter, and diameter orthogonal to it of the alteration in the EZ – hypo‐ and/or hyperreflective areas—were measured by two independent readers using integrated measurement tool. In cases without distinctive loss of EZ, the hyperreflective area visible on OCT was considered as the presumed area of EZ alteration.

### 2.1. Statistical Methods

Data were coded in Excel and analyzed with SPSS version 23. Descriptive statistics such as mean and standard deviations were computed.

Differences of largest diameter, orthogonal, area and BVA logMAR between the last and the first visits were computed. Non‐parametric paired Wilcoxon test was applied to investigate the decrease of measurements of EZ alteration and BVA with time. Two‐way mixed intraclass correlation coefficient was used for evaluation of inter‐reader reliability. The number of days between both measurements was computed. Non‐parametric Spearman correlation investigated the association between the numbers of days and the decrease of EZ alteration and BVA parameters.

Results with *p*‐value less than 5% were interpreted as statistically significant.

## 3. Results

An overview of patient's characteristics is given in Table 1. Patient's involved eyes showed a mean BVA of 20/36 (logMAR 0.25, SD ± 0.35) at first presentation and a mean BVA of 20/30 (logMAR 0.17 SD ± 0.37) at last follow-up.

**Figure 1:**
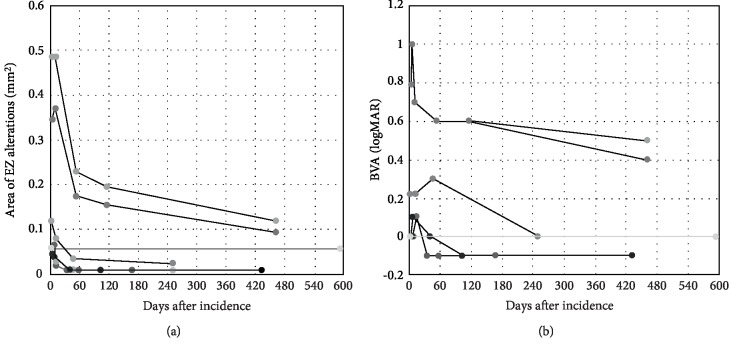
Plot of (a) area of ellipsoid zone (EZ) alterations (averages of both readers) and (b) best visual acuity (BVA) over time for all patients/eyes. Most of EZ alteration reduction and improvement of BVA occurred within first two month. Correlation between size of EZ alterations and BVA was significant (0.855, *p* < 0.001).

**Figure 2:**
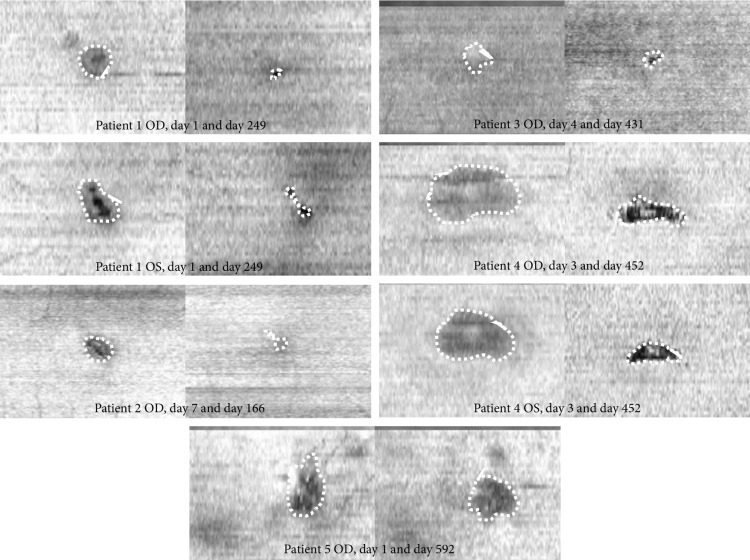
En face OCT of all patients at first and last visit. Patient 1 to 3 suffered laser injuries, patients 4 to 6 suffered solar maculopathy. Areas of EZ alterations are indicated by the dotted lines.

Mean area of EZ alterations was 0.12 mm^2^ (SD ± 0.10) at first presentation and 0.07 mm^2^(SD ± 0.08) at last follow-up for reader one (R1) and 0.21 mm^2^ (SD ± 0.22) and 0.05 mm^2^ (SD ± 0.04) for reader two (R2), inter‐reader correlation (including all follow ups) was 0.914. Average follow-up time was 372 (range 161–592) days. Statistical analysis showed a mean increase in BVA of approximately one line in Snellen (logMAR −0.17 SD ± 0.37, *p* = 0.027) and a mean decrease in the area of EZ alterations of 0.09 mm^2^ (SD ± 0.06, *p* = 0.018) comparing baseline examination and last follow-up for R1 and 0.16 mm^2^ (SD ± 0.19, *p* = 0.027) for R2. Further, the largest diameter of the EZ alterations as well as the diameter orthogonal to this showed significant reduction from first to last follow-up for both R1 and R2 (*p*‐values 0.018–0.028, inter‐reader correlations 0.966–0.968). All patients showed a decrease in the EZ alteration measurements and six out of seven eyes showed an increase in BVA, whereas the seventh eye showed a stable BVA of 20/20 (Figures 1 and 2).

Correlation of area of EZ alteration and BVA was significant (0.852, *p* < 0.001 for R1; 0.859, *p* < 0.001 for R2), using Spearman's rho. Most improvement in both EZ alterations and BVA occurred within first 2 months.

### 3.1. Report of Cases

**Figure 3:**
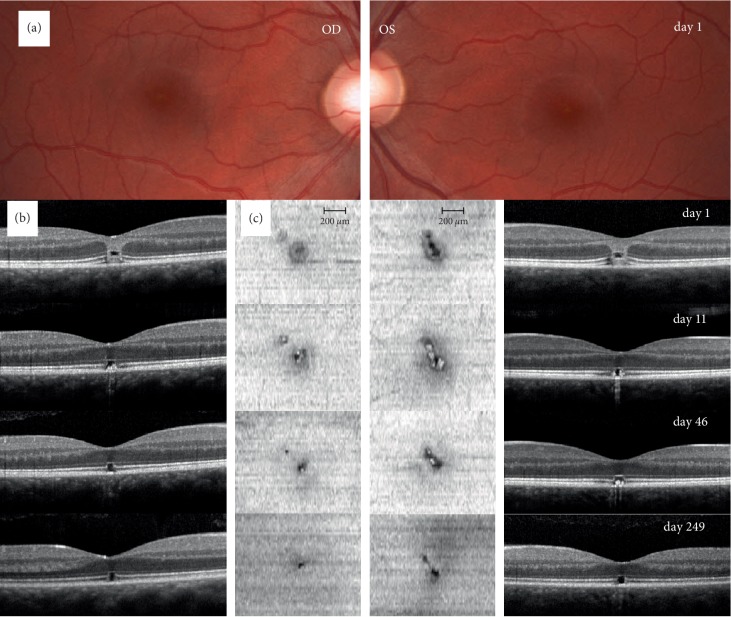
Regeneration of the ellipsoid zone in a patient with laser-induced maculopathy. (a) Fundus photography on day 1 after the accident showing small central lesion in both eyes of patient 1. (b) B‐scan OCT images through the fovea of the right and left eye depicts a hyperreflective band reaching from the retinal pigment epithelium (RPE) to the outer plexiform layer extending slightly to the perifoveal region (assumingly following the Henle fibers) and a hyporeflective zone expanding from the ellipsoid zone (EZ) to the external limiting membrane (ELM) in both eyes on day 1. It also showed thickened subfoveal outer retinal layers and a hyporeflective zone expanding from the EZ to the ELM. These findings diminish over follow-up (day 11, 46, and 249). Last follow-up on day 249 showed only a distinct hyporeflective zone in the outer retinal layers, boarded by intact ELM and RPE layers. (c) En face OCT images on the level of the EZ demonstrating a regeneration of the EZ alterations over time in both eyes. Early images show a central hyporeflective oval zone surrounded by a distinct hyperreflective ring and again a faint, fuzzily bounded hyporeflective zone. Late images show a pronounced hyporeflective defect in the EZ, decreasing in size over follow-up. Note the inhomogeneous choroidal hypertransmission as an indirect sign of incomplete RPE and outer retina damage visible on days 11 and 46 in both eyes [10].

Patient 1 (Figure 3) was a 23‐year‐old male who looked directly into a green laser pointer light with each eye individually, presumably out of curiosity. He presented himself one day after the incident with vision loss and central scotoma in both eyes. BVA at first consultation was 20/33 in both eyes. Anterior segment examination was normal. Dilated ophthalmoscopic examination showed a central yellow spot in both eyes (Figure 3(a)). A hyperreflective band reaching from the RPE to the outer plexiform layer as well as a hyporeflective zone expanding from the EZ to the ELM (Figure 3(b)) could be observed on OCT. On last follow-up a distinct hyporeflective zone in the outer retina, boarded by intact ELM and RPE was noted. Early en face OCT on the level of the EZ showed a hyporeflective oval zone surrounded by an inner distinct hyperreflective ring and an outer faint hyporeflective zone (Figure 3(c)). Follow-up of en face OCT showed a pronounced hyporeflective defect in the EZ, reducing in size over several months. BVA dropped to 20/40 in both eyes at week six and improved to 20/20 in both eyes at last consultation 8 months after the incident.

**Figure 4:**
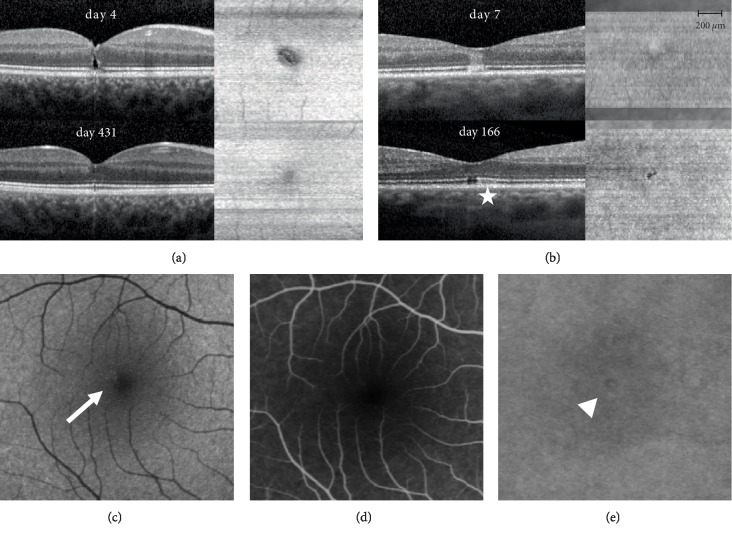
B‐scan and en face OCT images of patient 2 and 3 (a and b) and multimodal imaging data including autofluorescence imaging (c), fluorescein (d) and indocyanine green (e) angiography of patient 2. (a) OCT B‐scan on day 7 through the foveal center shows a lammelar hole reaching up to the outer nuclear layer; En face OCT shows an EZ alteration boarded by a hyperreflective ring; On day 166 (last follow-up) closure of the lamellar hole and near complete regeneration of the EZ. An inhomogeneous choroidal hypertransmission can be detected until the last follow-up (*star*). (b) B‐scan OCT on day 4 through the foveal center demonstrates a typical hyperreflective band in the outer retina reaching from the RPE to the outer plexiform layer; there was no distinct hyporeflective zone and no thickening of the outer retinal layers. One month after the incident, B‐scan showed a distinct hyporeflective defect in the ellipsoid zone (EZ), which persisted until last follow-up on day 431. En face OCT on day 4 shows a mild hyperreflective circle, partially surrounded by a scantly hyporeflective zone area; on day 431 a clearly demarcated EZ loss can be observed. (c) Autofluorescence (Heidelberg Spectralis HRA+OCT, excitation at 488nm, barrier filter wavelength of 500nm) showed a hyperautofluorescent zone at first presentation (seven days after the incidence) in the impact region (*arrow*). (d) Fluorescein angiography (ten minutes after injection) ten days after the incidence did not show any leakage or staining. (e) Indocyanine‐green (ICG) angiography (ten minutes after injection) did show minimal irregular hyper‐ and hypocyanescence in the region of impact (*arrowhead*).

Patient 2 was a 19‐year‐old woman who had looked directly into a laser show light projector of unknown strength on holidays abroad and experienced a small central scotoma in her right eye (Figure 4(a)). OCT showed a lamellar macular hole which resolved completely within 2 months. There was a small area of hyperautofluorescence at first presentation in the impact region (Figure 4(c)). Fluorescein angiography 10 days after the incident did not show any leakage or staining (Figure 4(d)). Indocyanine‐green (ICG) angiography at the same time point did show minimal irregular hyper‐ and hypocyanescence in the region of impact in the late frames (Figure 4(e)). BVA first dropped to 20/25 ten days after the incident (while EZ alteration was already decreasing in size) and then improved to 20/15 after one month.

Patient 3 (Figure 4(b)) was a 38‐year‐old male who watched a solar eclipse without any protection, but he closed the eyes alternatively. He presented himself 4 days after the incident due to ongoing decreased vision in the right eye and small central scotoma. BVA at first consultation was 20/20 in both eyes. Anterior segment examination was normal. Dilated ophthalmoscopic examination of the right eye showed central pigment alteration. A hyperreflective band in the central fovea reaching from the RPE to the outer plexiform layer was noted on B‐scan OCT. A hyporeflective zone in the outer retinal layers was not present at baseline, but appeared later during follow-up. En face OCT at the level of the EZ showed a mild hyperreflective ring, presumed as area of EZ alteration. A distinct hyporeflective defect in the EZ layer was seen one month after the incident, which then reduced in size but persisted until last follow-up. BVA dropped by one line to 20/25 in the right eye one week after the incident, improved again to 20/20 at one month and to 20/15 at 14 weeks and thereafter.

**Figure 5:**
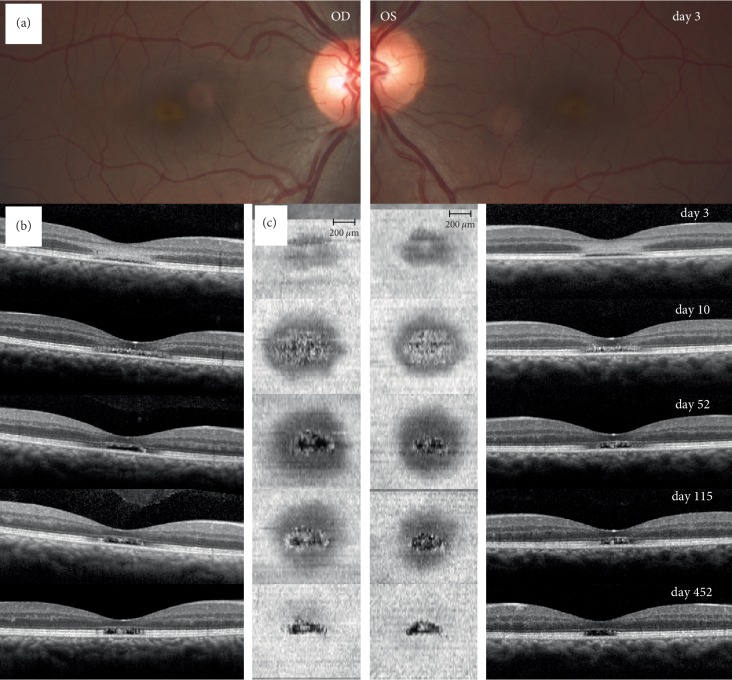
Fundus photography, B‐scan, and en face OCT images of a patient with solar maculopathy. (a) Fundus photography on day 3 after the accident shows central pigment alterations in both eyes of patient 4. (b) B‐scan OCT images through the fovea show disruption of the retinal pigment epithelium (RPE) and the ellipsoid zone (EZ) as well as a hyperreflective band reaching from the outer nuclear layer to the outer plexiform layer (whereas the external limiting membrane (ELM) seemed to be intact) in both eyes on day 3, diminishing in the follow-up images (days 10, 52 and 115), while presenting hyperreflective granular appearances at the level of the EZ. Last follow-up 452 days after the incidence showed a smaller, clearly delineated EZ alteration with persistent hyperreflective granular appearances boarded by intact ELM and RPE layers. (c) En face OCT at the level of the EZ on day 3 shows a hyporeflective defect surrounded by a hyperreflective ring in both eyes. On day 10, the hyporeflective defect expands to the region where the hyperreflective ring was seen and a central defect with granular appearances comes to light. Follow ups on days 52 and 115 show a regression of the granular appearing central defect. Last follow-up 452 days after the incidence shows a smaller, clearly delineated EZ loss suggesting a partial, centripetal regeneration of the EZ alteration over time in both eyes.

Patient 4 (Figure 5) was a 41‐year‐old male with a history of schizophrenia which was currently treated with escitalopram, a selective serotonin reuptake inhibitor, not known to have any effect on phototoxicity. He presented himself three days after sudden onset of vision loss and central scotoma in both eyes. On the day before onset of symptoms, he participated in a street parade. He denied sun gazing or drug abuse, but reconstruction of the events was difficult. At first consultation, BVA was 20/123 in both eyes. Anterior segment examination was normal. Dilated ophthalmoscopic examination showed a clearly yellow area at the fovea with a reddish centre in both eyes (Figure 5(a)). B‐scan OCT demonstrated disruption of the RPE and the EZ and a wide hyperreflective band reaching from the outer nuclear layer to the outer plexiform layer (Figure 5(b)), similar to patient 1. En face OCT at the level of the EZ showed a hyporeflective defect surrounded by a hyperreflective ring (Figure 5(c)), which evolved into a central alteration with granular appearances and expanding in size before regressing to a clearly delineated EZ loss. BVA first dropped to 20/200 in both eyes 4 days after the incident, improved again to 20/100 in both eyes at 10 days, to 20/80 in both eyes at 2 months and was 20/50 in right and 20/60 in left eye at last follow-up. Initial presentation and evolution let presume a solar maculopathy.

Patient 5 was a 72‐year‐old woman experiencing a paracentral scotoma in the right eye, after observing a solar eclipse. OCT en face imaging showed only slight EZ regeneration (although followed for 592 days). BVA was always 20/20.

## 4. Discussion

Laser‐induced and solar maculopathies are relatively rare events. These injuries often result in EZ alterations and decreased BVA. This case series demonstrates a significant reduction of EZ alterations over time after such an injury and that BVA is strongly correlated with the size of EZ loss.

In most patients, hyporeflective cavities on the level of the RPE and/or the EZ with an overlying hyperreflective band reaching up to the outer plexiform layer, appearing to follow the Henle fibers, could be detected. This is consistent with other reports [5, 6, 11] which described the primary defect site to be the RPE with involvement of the outer photoreceptor segments by photochemical and/or photothermal reactions in extended injuries [1, 12]. In laser‐induced retinal injuries in zebra fish the early hyperreflectivity seen in OCT histologically correlates to an early edema, which is followed by disorganization of photoreceptors and loss of nuclei in the outer nuclear layer after one day [13], keeping in mind that OCT images are based on tissue reflectivity and not on tissue or cell types (while reflectivity is amongst other things depending on the complex refractive index of the cellular and extracellular components involved and light scattering) [14, 15]. With en face OCT, we could depict the total two‐dimensional alterations of the EZ and carry out a quantitative analysis of the EZ reformation. All our patients showed significant reduction of EZ alteration.

Comparing solar maculopathy and laser injury, very similar findings were observed, except in one patient with a lamellar macular hole due to a laser injury, which was not seen in any of the solar maculopathy cases. This finding is most likely caused by the higher energy level in lasers compared to sun light: Local rise of temperature of the retina when sun gazing was calculated to be only about 2°, far below the 10° needed to cause thermal damage [16]. Solar maculopathy is most likely caused by photochemical damage, which involves cellular damage (ultimately resulting in cell death) by supercharged molecules. In laser light, energy can be far higher and therefore cause thermal (which involves direct corruption of the structure of proteins) or even disruptive damage [17, 3]. No qualitative difference could be seen in reformation of the EZ and subgroups were too small for statistical comparison.

Mechanism of the reformation of the EZ is unclear. There are multiple reports of total or partial EZ reformation in other retinal pathologies primarily involving photoreceptors such as in multiple evanescence white dot syndrome, acute macular neuropathy, and Vogt‐Koyanagi‐Harada disease [18–20]. Recent evaluations found that regeneration of only about 50% of photoreceptors might result in normal sensitivity to light [21]. However, reformation on the cellular level is not well studied. Rabbits and mouse models show migration of photoreceptors from unaffected areas, thereby replacing the necrotic photoreceptors and building new functional synaptic contacts with unaffected overlying bipolar cells and light responsive retinal ganglion cell density recovers within 2 months [22, 23]. If similar cellular regeneration occurs in the human retina has yet to be investigated.

In our cases, most reformation of the EZ and recovery of visual acuity occurred within 2 months. BVA correlated with the extent of EZ alteration at any given time, but prediction of the visual outcome in a given injury at baseline is not possible. Clinical reports of laser injuries in children suggested a subdivision into “severe” injuries with poor visual outcome and “light” injuries with good visual outcome [24, 25]. Our cohort does support this subdivision, with only patient 4 presenting a severe injury (in both eyes) with only partial recovery and all others recovering to a BVA of 20/20 or better.

One major limitation of this study is that SD OCT can only visualize the EZ but neither the photoreceptors themselves nor their synaptic contacts; therefore, we can only describe the reformation of the EZ, which is in the end an artificial imaging effect solely existing on OCT, while the events on cellular level remain unknown. As in many other reports of solar maculopathies or laser injuries, the small number of patients limits the statistical significance of the results.

## 5. Conclusions

In our series of seven eyes, we demonstrated that there is reformation of the EZ on OCT in solar maculopathy as well as in laser injury to the central retina over time. Pattern of injury is very similar in both entities, where a laser injury may present additional risk of disruptive damage, in concordance with earlier publications. Further, we found a significant correlation of BVA and the extent of EZ loss at a given time.

## Figures and Tables

**Table 1:** Patients characteristics, diagnosis, and visual acuity outcome.

Patient no.	Age (years)	Interval between exposure and presentation (days)	Eye involved	Diagnosis	Initial visual acuity (Snellen)	Last follow‐up (days)	Last visual acuity (Snellen)
1	23	1	OD	Laser‐induced maculopathy	20/33	249	20/20
			OS	Laser‐induced maculopathy	20/33	24	20/20
2	19	7	OD	Laser‐induced maculopathy	20/20	16	20/16
3	38	4	OD	Solar maculopathy	20/20	431	20/16
4	41	3	OD	Solar maculopathy	20/123	45	20/50
			OS	Solar maculopathy	20/123	45	20/60
5	72	1	OD	Solar maculopathy	20/20	592	20/20

## Data Availability

The en face images and the raw measurements used to support the findings of this study are available from the corresponding author upon request.

## Supplementary Materials

Supplementary Materials Figure 6: macular autofluorescence images of patient 4; day 3, day 10 and day 115 (Heidelberg Spectralis HRA+OCT, excitation at 488 nm, barrier filter wavelength of 500 nm). Images show extensive areas of hypoautofluorescence on day 3 and day 10 after the incident. Images on day 115 show normalization of autofluorescence.
