# Supplementary material for: En Face Optical Coherence Tomography Imaging Ellipsoid Zone Regeneration in Laser-Induced and Solar Maculopathies
Source: Case Rep Ophthalmol Med. 2019 Nov 21;2019:3849871. doi: 10.1155/2019/3849871 (PMC6925683; doi:10.1155/2019/3849871)
Supplement: Supplementary Materials — Figure 6: macular autofluorescence images of patient 4; day 3, day 10 and day 115 (Heidelberg Spectralis HRA+OCT, excitation at 488 nm, barrier filter wavelength of 500 nm). Images show extensive areas of hypoautofluorescence on day 3 and day 10 after the incident. Images on day 115 show normalization of autofluorescence. [file 3849871.f1.pdf]

Supplementary file

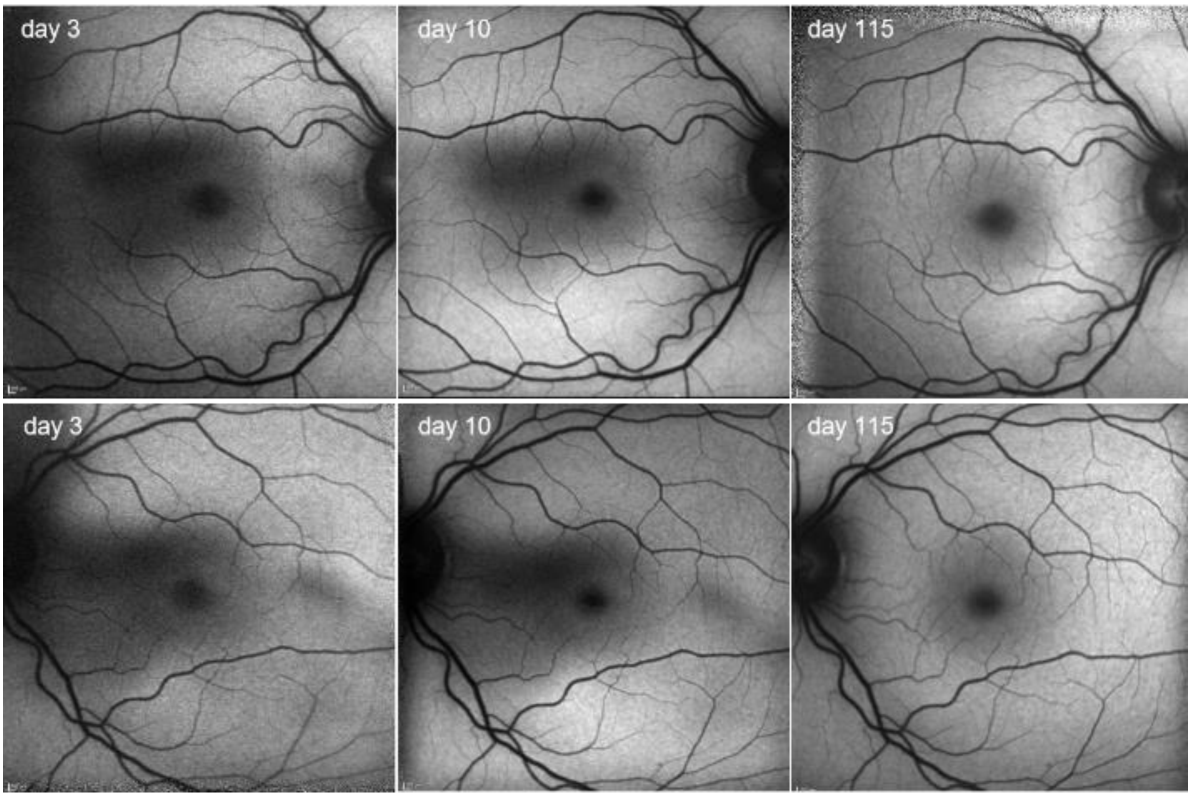

Figure 6: Fundus autofluorescence images of patient 4 obtained with a confocal scanning laser ophthalmoscope (cSLO) demonstrating substantially diminishes autofluorescence in the parafoveal areas in both eyes on day 3 after the incident. Images on day 115 show apparently normal autofluorescence (excitation wavelength of 488nm and an emission filter of 500nm was used; macular pigment markedly reduces autofluorescence and therefore the foveal aspects cannot be evaluated).
